# Titanium dioxide in our everyday life; is it safe?

**DOI:** 10.2478/v10019-011-0037-0

**Published:** 2011-11-16

**Authors:** Matej Skocaj, Metka Filipic, Jana Petkovic, Sasa Novak

**Affiliations:** 1 Jožef Stefan Institute, Department for Nanostructured Materials, Ljubljana, Slovenia; 2 National Institute of Biology, Department for Genetic Toxicology and Cancer Biology, Ljubljana, Slovenia

**Keywords:** titanium dioxide, nanoparticles, toxicity, applications, safety

## Abstract

**Background:**

Titanium dioxide (TiO_2_) is considered as an inert and safe material and has been used in many applications for decades. However, with the development of nanotechnologies TiO_2_ nanoparticles, with numerous novel and useful properties, are increasingly manufactured and used. Therefore increased human and environmental exposure can be expected, which has put TiO_2_ nanoparticles under toxicological scrutiny. Mechanistic toxicological studies show that TiO_2_ nanoparticles predominantly cause adverse effects via induction of oxidative stress resulting in cell damage, genotoxicity, inflammation, immune response etc. The extent and type of damage strongly depends on physical and chemical characteristics of TiO_2_ nanoparticles, which govern their bioavailability and reactivity. Based on the experimental evidence from animal inhalation studies TiO_2_ nanoparticles are classified as “possible carcinogenic to humans” by the International Agency for Research on Cancer and as occupational carcinogen by the National Institute for Occupational Safety and Health. The studies on dermal exposure to TiO_2_ nanoparticles, which is in humans substantial through the use of sunscreens, generally indicate negligible transdermal penetration; however data are needed on long-term exposure and potential adverse effects of photo-oxidation products. Although TiO_2_ is permitted as an additive (E171) in food and pharmaceutical products we do not have reliable data on its absorption, distribution, excretion and toxicity on oral exposure. TiO_2_ may also enter environment, and while it exerts low acute toxicity to aquatic organisms, upon long-term exposure it induces a range of sub-lethal effects.

**Conclusions:**

Until relevant toxicological and human exposure data that would enable reliable risk assessment are obtained, TiO_2_ nanoparticles should be used with great care.

## Introduction

Titanium dioxide (titania, TiO_2_) is chemically inert, semiconducting material that also exhibits photocatalytic activity in the presence of light with an energy equal to or higher than its band-gap energy. These characteristics offer a wide range of applications. For these reasons, and because of the relatively low price of the raw material and its processing, titania has gained widespread attention over recent decades.

TiO_2_ has been classified in humans and animals as biologically inert^1^,^2^, and is widely considered to be a “natural” material, which at least partially contributes to its relatively positive acceptance by the public. In fact, most TiO_2_ has been synthesized from the mineral illmenite, FeTiO_3_, using the “sulphate” or “chloride” process for nearly 100 years. The annual worldwide production of titania powder in 2005 has been estimated to be around 5 million tons^3^, provoking the question as to its abundance in the environment. The proportion of nano-sized titania is estimated to have been approximately 2.5 % in 2009, increasing to 10 % by 2015^4^, with an exponential increase over the past decade.

During recent decades, TiO_2_ powders have begun to appear in many applications, mainly due to their ability to confer whiteness and opacity on various products, such as paints, papers and cosmetics. Its high technological attractiveness originates from its light-scattering properties and very high refractive index, which mean that relatively low levels of the pigment are required to achieve a white, opaque coating. The range of light that is scattered depends on the particle size. Numerous technological improvements, based on nano-sized TiO_2_, have been introduced that enable its use for antifogging and self-cleaning coatings on glass, for building facades, in confectionary, in the plastics industry, and so on. Furthermore, TiO_2_ is accepted as a food and pharmaceutical additive.^5^ In the United States it is included in the Food and Drug Administration (FDA) Inactive Ingredients Guide for dental paste, oral capsules, suspensions, tablets, dermal preparations and in non-parenteral medicines.

The increasing production of nano-sized TiO_2_ powder has led to growing concerns about the consequences of exposure of humans and the environment.^6^ In the present paper we review and discuss the latest findings on potential hazard of exposure to nano-sized TiO_2_ for humans and environment, in regard to the particle size and the crystal structure of TiO_2_, the route of exposure as well as the effect of ultraviolet (UV) irradiation-induced photocatalysis.

## Chemical and physical properties of TiO_2_ nanoparticles

Nanoparticles (NPs) are generally defined as particles having at least one dimension smaller than 100 nm. Accordingly, particles with different morphologies, from equi-axial shapes, whiskers, and nano-tubes to nanorods, need to be considered. Although micron-sized and nano-sized TiO_2_ powders are, in general, chemically identical, due to their significantly higher specific surface area, nano-powders may exhibit physical and chemical properties that differ from those of the coarser grades, and so should not be treated in the same way. In a recent paper^7^ the size-dependent properties of a variety of inorganic NPs were reviewed and it was suggested that they are likely to be of concern due to the appearance of unique properties when they have diameters of ≤ 30 nm. In this size range, many particles undergo dramatic changes in behaviour that enhance their interfacial reactivity. While less than 20 % of the constituent atoms are at the surface of 30 nm NPs, approximately 35–40 % of the atoms are localized at the surface of a 10 nm particle.

**FIGURE 1:**
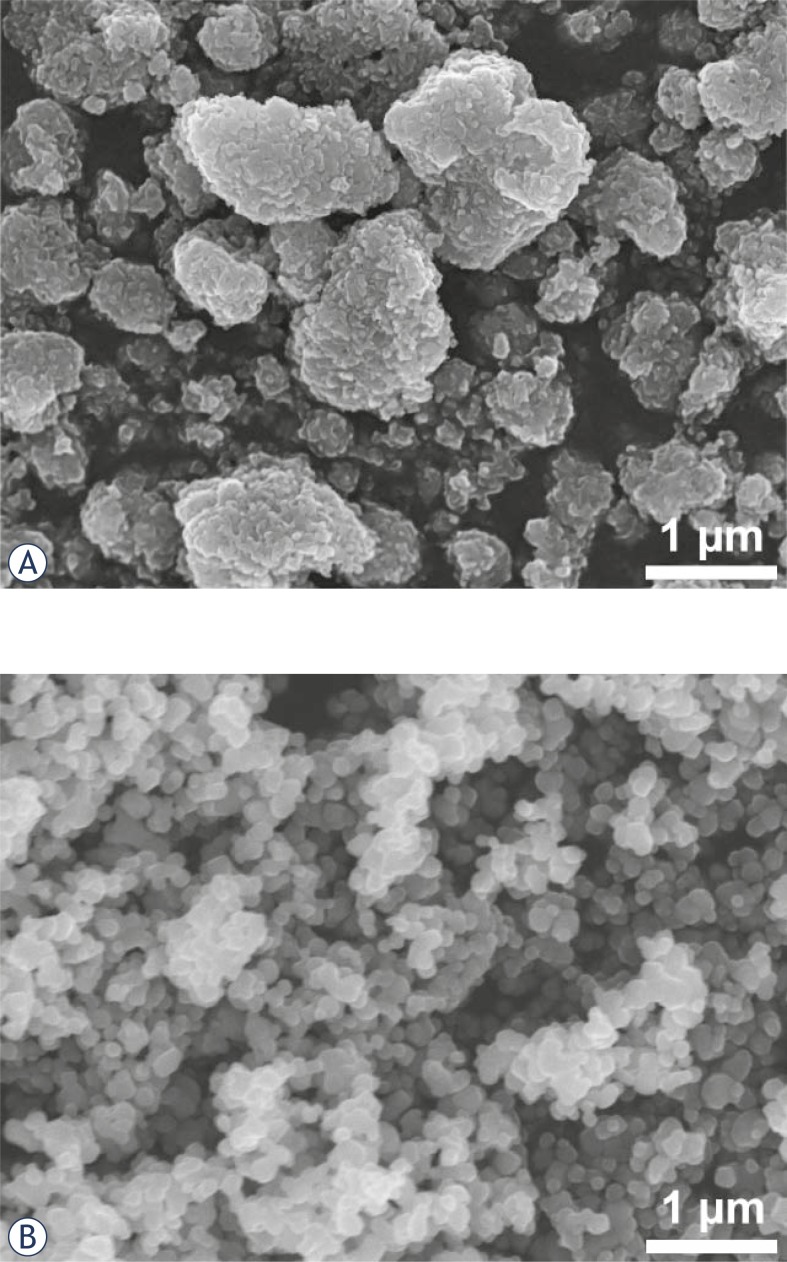

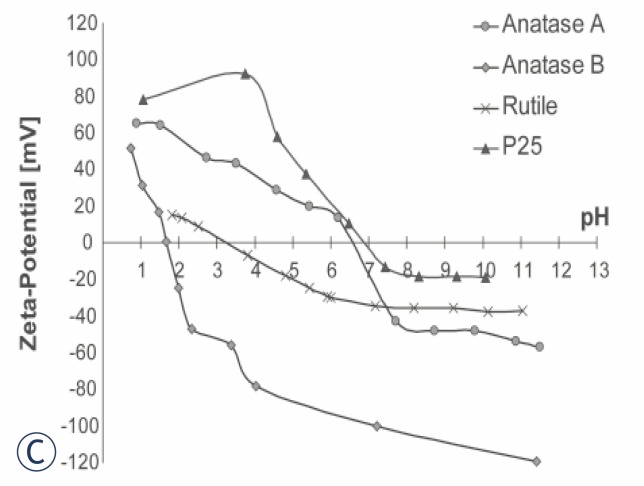
Field emission electron micrographs of different TiO_2_ powders: A) Anatase A (Sigma 637254), B) Anatase B (Sigma T8141); C) Zeta-potential of these two powders, Rutile (Sigma 637262) and P25 (Degussa).

In practice, it is difficult to draw a clear borderline between nano- and submicron-sized particles. Submicron-sized powders always contain a certain proportion of nano-sized particles and, conversely, NPs tend to associate to form relatively strongly bonded aggregates (Figure 1A) or soft agglomerates (Figure 1B). The latter can usually be disintegrated easily in a liquid; however, their dispersion depends strongly on the zeta-potential. As illustrated in Figure 1C, the zeta-potential of TiO_2_ powders may differ significantly over a wide range of pH values. The reported isoelectric points for TiO_2_ powders range from pH 3.5 to 8 8 which may greatly affect the bioavailability in the region of physiological pH values. The effective size of particles and their zeta-potential have been neglected almost completely in most of the studies of the interaction of TiO_2_ NPs with biological systems.

Crystalline TiO_2_ occurs naturally in three polymorphs – anatase, rutile and brookite – among which rutile is the most stable. A powder with an average particle size of 230 nm scatters visible light, while its counterpart, with an average size of 60 nm, scatters UV light and reflects visible light. Under UV, TiO_2_ exhibits photocatalytic activity, which is a consequence of the electronic structure of the titania, and is, to a large extent, more characteristic of anatase than of rutile and brookite. In the presence of light with energy equal to or higher than the TiO_2_ band-gap energy, an electron is promoted from the valence band to the conduction band, leaving behind a positive hole. The extrapolated optical absorption gaps of anatase and rutile are 3.2 and 3.0 eV at room temperature, which correspond to wavelengths of around 413 nm and 387 nm. Consequently, the photo-activation of nano-TiO_2_ can be achieved by irradiation with UV-A, B and C, visible, fluorescent light, and X-ray radiation. The photocatalytic activity results in formation of highly reactive radicals, that are capable of reacting with most of the surrounding organic substances.^9^–^12^

## Mechanisms of TiO_2_ NPs toxicity

As already discussed, the physicochemical properties of particles depend on their size, so that, at the nanometre level, the material is chemically more reactive. This can be exploited as a desirable property, *e.g.*, as a catalyst. However, at the same time, the material may possess biological activities that can be either desirable (*e.g.,* carrier capacity for therapeutics, penetration of cellular barriers for drug delivery) or undesirable (*e.g.,* toxicity, induction of oxidative stress or cellular dysfunction), or a mix of the two.

### Cellular uptake of TiO_2_ NPs

From a toxicological point of view the important characteristics of NPs are their size, surface area, surface chemistry and charge, crystallinity, shape, solubility and agglomeration/aggregation state. Surface groups may render NPs hydrophilic or hydrophobic, lipophilic or lipophobic, catalytically active or passive. Cellular uptake, subcellular localization, and ability to cause toxic effects depend on these properties of NPs.^13^ The two main pathways of NP uptake in the cell are active uptake by endocytosis, and passive uptake by free diffusion. Phagocytosis is an actin-dependent, endocytic mechanism, typical of “professional” phagocytes like macrophages. Geiser *et al*.^14^ reported that, in rats exposed to TiO_2_ powders by inhalation, alveolar macrophages effectively cleared micron-sized (3–6 μm) but not nano-sized (20 nm) TiO_2_ particles. This is important, since phagocytes generally remove particulate matter >500 nm 15 and, as they are unable to phagocytose smaller particles, the latter are retained in the tissue, leading to a sustained burden on other tissues and cells. It was demonstrated that the uptake of 50 nm nano-TiO_2_, by endocytosis with alveolar A549 epithelial cells, was limited to aggregated particles.^16^ After inhalation exposure of rats to TiO_2_ NPs, free particles were found within the cytoplasm of epithelial and endothelial cells and fibroblasts.^17^ Rothen-Rutishauser *et al.*^18^, used an *in vitro* airway wall model, and found membrane-bound aggregates (>200 nm) of TiO_2_ as well as smaller unbound aggregates within the cell cytoplasm. In an *in vitro* study Kocbek *et al.*^19^ demonstrated the endocytotic uptake of 25 nm-sized anatase TiO_2_ by human keratinocytes. They observed highly aggregated NPs within early and late endosomes and in amphisomes, confirming endocytotic uptake. Experiments with red blood cells, which lack phagocytic receptors^18^, revealed that TiO_2_ NP aggregates smaller than 200 nm are able to enter red blood cells, while larger particles were only found attached to the cell’s surface. Xia *et al*.^20^ showed that fluorescence-labelled TiO_2_ NPs (11 nm) were taken up and localized in late endosomal and caveolar compartments in phagocytic RAW 246.7 and lung endothelial BEAS-2B cells.

### Oxidative stress induced by TiO_2_ NPs

Oxidative stress is thought to be a key mechanism responsible for adverse biological effects exerted by NPs.^21^,^22^ The role of oxidative stress in TiO_2_-induced adverse effects has been confirmed by evidence that it induces an increase in reactive oxygen species (ROS) production and oxidative products (*i.e.*, lipid peroxidation), as well as the depletion of cellular antioxidants.^23^–^29^

TiO_2_ mediates oxidative stress under UV irradiation as well as without it. Uchino *et al*.^30^ showed that, under UV irradiation, the TiO_2_ NPs of different crystalline structures and sizes produces different amounts of hydroxyl radicals, and that cytotoxicity against Chinese hamster ovary cells correlates with the production of radicals. Dodd and Jha^31^ confirmed that hydroxyl radicals are the primary damaging species produced by UV irradiated nano-sized TiO_2_, and react to give carboxyl radicals. A number of studies have shown photo-activated anatase TiO_2_ to induce higher cytotoxicity and genotoxicity than similarly activated rutile TiO_2_. These differences could arise from the fact that anatase particles possess a wider absorption gap and a smaller electron effective mass, resulting in the higher mobility of the charge carriers and the greater generation of ROS. On the other hand, there is evidence that TiO_2_ also induces ROS formation and the associated adverse effects in the absence of photo-activation. For instance, Gurr *et al*.^24^ found that anatase TiO_2_ NPs and mixtures of anatase and rutile TiO_2_ NPs induced oxidative damage in human bronchial epithelial (BEAS-2B) cells, and Petkovi *et al*.^32^ reported that in human hepatoma cells (HepG2), non-irradiated anatase nano-TiO_2_ induced significantly higher levels of intracellular ROS than the corresponding rutile-TiO_2_, and only anatase nano-TiO_2_ caused oxidative DNA damage. Recently, Petkovi *et al*.^33^ compared cytotoxicity and genotoxicity of non-irradiated and UV pre-irradiated anatase TiO_2_ of two sizes (<25 nm and >100 nm). They showed that non-irradiated TiO_2_ particles did not affect survival of the cells; they caused slight increase in number of DNA strand breaks, while only TiO_2_ NPs caused increase in oxidative DNA damage. After pre-irradiation with UV both sizes of anatase TiO_2_ particles reduced cell viability, induced DNA strand breaks and oxidative DNA damage. This is an important finding that, for the first time, showed that photo-activated TiO_2_ particles retained higher cytotoxic and genotoxic potential also when UV irradiation was discontinued and that it was not particle size dependent.

ROS are also important signalling modulators, therefore exposure of cells to NPs may, via elevated ROS formation, affect cellular signalling cascades that control processes such as cell proliferation, inflammation and cell death.^34^ The role of oxidative stress in TiO_2_-induced inflammation has recently been confirmed by Kang *et al*.35 In the mouse peritoneal macrophage cell line RAW 246.7 exposed to nano-TiO_2_, ROS production was associated with the activation of pro-inflammatory cascade, as indicated by extracellular signal-regulated kinases ERK1/2 phosphorylation, tumour necrosis factor TNFα production and macrophage inflammatory protein MIP-2 secretion.

Taken together, these studies indicate that the high level of oxidative stress that is related to an exposure to a high concentration of TiO_2_ NPs leads to cell damage-associated responses, whereas at moderate levels of oxidative stress, inflammatory responses may be stimulated by the activation of ROS-sensitive signalling pathways.

### Genotoxicity of TiO_2_ NPs

Several studies show that nano-TiO_2_ induces ge-notoxic effects, including DNA damage, and micronuclei formation that is indicative of chromosomal aberrations in different cell lines^32^, ^35^–^38^ The studies also showed that genotoxic effects elicited NPs strongly depended upon their size by TiO_2_ and form. For instance, Gurr *et al*.^24^ showed that NPs up to 20 nm in size induced an anatase TiO_2_ increase in micronuclei formation, while 200 nm did not. Zhu *et al*.^39^ anatase or 200 nm rutile TiO_2_ demonstrated clear differences in the cytotoxicity and the extent of DNA strand scission, together with the formation of 8-hydroxy-2-deoxyguanosine (8-OHdG) adducts in isolated DNA, after a treatment with different types of TiO_2_ NPs in the order 10-20 nm anatase > 50-60 nm anatase > 50-60 nm rutile. At the molecular level it has been shown that the exposure of peripheral human lymphocytes to TiO_2_ NPs caused the activation of DNA damage check points and the accumulation of tumour suppressor protein p53, the main regulator of the cellular response to DNA damage.^40^ Exposure of human hepatoma HepG2 cells under similar conditions led to the elevated expression of tumor suppressor p53 mRNA and its downstream regulated DNA damage response genes (cyclin-dependent kinase inhibitor p21, growth arrest and DNA damage-inducible gene *GADD45a* and the E3 ubiquitin ligase *MDM2*).^32^

On the other hand, TiO_2_ NPs were devoid of mutagenic activity in microbial mutation assays (with *Salmonella typhimurium*) and in chromosomal aberration the in Chinese hamster ovary cells.^41^ Similarly, Theogaraj *et al*.^42^ reported that nano-sized TiO_2_ (eight different anatase and rutile forms) at concentrations up to 5 mg/ml did not induce any increase in the chromosomal aberration frequency in Chinese hamster ovary cells, in either the absence or the presence of UV light. However, in this study only a short-term, 3-hour, and no continuous (*i.e*., 20 hours) exposure, was performed.

In an early study Driscoll *et al*.^43^ reported that in rats intratracheal instillation of TiO_2_ NPs (100 mg/kg BW) induced increased HPRT mutation frequency in alveolar cells. They also showed that mutagenicity in alveolar cells was associated with inflammation. Trouiller *et al*.^44^ recently reported that oral exposure of mice to TiO_2_ NPs through drinking water (50–500 mg/kg BW/day for 5 days) induced oxidative DNA damage, micronuclei formation and γ-H2AX foci, the indicators of DNA double strand breaks. Since also high-level gene expression of pro-inflammatory cytokines was also observed, the authors suggested the inflammatory effects were responsible for the induction of genotoxic effects.

The *in vitro* and *in vivo* genotoxicity studies using different experimental models indicate that nano-TiO_2_ may cause genotoxic effects via secondary mechanisms that include oxidative stress and inflammation.^32^,^38^,^40^,^43^,^44^ However, there is some evidence that nano-sized TiO_2_ can locate in nuclei 17, and recently Li *et al*.^45^ reported the presence of anatase nano-sized TiO_2_ in DNA extracted from the liver of mice exposed intraperitoneally to these NPs (5–150 mg/kg BW/day for 14 days). The authors showed that Ti inserted between DNA base pairs or bound to DNA nucleotides, in such a way that it altered the conformation of the DNA and, at higher doses, caused DNA cleavage. These findings indicate that TiO_2_ may also induce genetic damage by a direct interaction with the DNA.

### Immunotoxic effects of TiO_2_ NPs

Depending on physicochemical properties of NPs, they are recognized and taken up by immune cells, such as macrophages, monocytes, platelets, leuko-cytes and dendritic cells, and can trigger an inflammatory response. In a human monoblastoid cell line (U937) exposure to TiO_2_ NPs induced apoptosis and necrosis in concentrations corresponding to those found in blood, plasma, or in tissues surrounding Ti implants 46. Palomäki *et al*.^47^ reported that rutile TiO_2_ NPs and silica-coated rutile TiO_2_ NPs induced the enhanced expression of a variety of proinflammatory cytokines in murine dendritic cells (bm-DC) and in murine macrophages (RAW 246.7). The particles were for dendritic cells more toxic than for macrophages. In dendritic cells nano-sized TiO_2_ led to an upregulation of maturation markers and activated the NLRP3 inflammasome, a multiprotein complex within the cytoplasm of antigen-presenting cells, leading to significant IL 1β-secretion. It was demonstrated for neutrophils that the short-term exposure of neutrophils to nano-anatase TiO_2_ induces changes in their morphology, indicating its potential to activate these cells, while longer exposure resulted in the inhibition of apoptosis and cytokine production, confirming that *in vitro* TiO_2_ exerts neutrophil agonist properties.

Immunomodulating effects after exposure to TiO_2_ NPs have been observed also in *in vivo* studies. Larsen *et al*.^48^ showed that in ovalbumin immunized mice intraperitoneal exposure to TiO_2_ NPs promoted a T-helper type 2 cells mediated dominant immune response with high levels of oval-bumin-specific immunoglobulins IgE and IgG1 in serum and influx of eosinophils, neutrophils and lymphocytes in bronchoalveolar lavage fluid. Airway inflammation and immune adjuvant activity in ovalbumin immunized mice was observed also after intranasal exposure to TiO_2_ NPs 48,49 indicating that airborne exposure to TiO_2_ NPs may induce respiratory allergy, where the possible mechanism could be an adjuvant-like activity of NPs on allergic sensitization. Associated with the impairment of the immune response, recently Moon *et al*.^50^ showed that the intraperitoneal exposure of mice to TiO_2_ NPs enhanced the growth of subcutaneously implanted B16F10 melanoma through the immunomodulation of B- and T-lymphocytes, macrophages, and natural killer cells.

### Neurotoxic effects of TiO_2_ NPs

It has been reported that inhaled NPs can translocate to the central nervous system through the olfactory pathway^22^ and by crossing the blood-brain barrier.^51^,^52^ *In vitro* studies of non-irradiated TiO_2_ NPs (Degussa P25) showed that they cause oxidative stress in the brain microglia BV2 cell line 53 that was associated with the up-regulation of genes involved in the inflammation, apoptosis, and the cell cycle, and down-regulation of genes involved in energy metabolism.^25^ While Degussa P25 NPs stimulated ROS formation in BV2 microglia, they were nontoxic to isolated N27 neurons. However, in complex brain cultures the Degussa P25 particles rapidly damaged neurons, plausibly through microglial generated ROS. In contrast, Liu *et al.*^54^ reported that, in the neuronal cell line PC12, exposure to nano-TiO_2_ induced dose-dependent oxidative stress and apoptosis that was partly prevented by pre-treatment with a ROS scavenger. Surprisingly, it was shown recently 55 that TiO_2_ NPs (rutile TiO_2_ coated with SiO_2_; 80–100 nm) might be an inducer of the differentiation of (mouse) neural stem cells towards neurons. These results indicate that the responses may be cell-type dependent and oxidative stress-mediated.

Recently Scuri *et al.*^56^ reported that inhalation exposure of newborn (2-day-old) and weanling (2-week-old), but not adult, rats to TiO_2_ NPs (12 mg/m^3^; 5.6 h/day for 3 days) up-regulates the expression of lung neurotrophins, key regulatory elements of neuronal development and responsiveness that play a critical role in the pathophysiology of childhood asthma. The effect was associated with the development of airway hyperreactivity (AHR) and mild airway inflammation. These results suggest the presence of a critical window of vulnerability in the earlier stages of lung development, which may lead to a higher risk of developing asthma.

## TiO_2_ NPs in everyday life

Nano-sized TiO_2_ in various forms is used widely in everyday life in a variety of products, such as anti-fouling paints, household products, plastic goods, medications, cosmetics, sunscreens, pharmaceutical additives and food colorants, and many new applications are under development or already in pilot production. In the following sections we consider the main entry ports of nano-sized TiO_2_ into the human body and potential adverse effects.

### Dermal exposure to TiO_2_ NPs

#### TiO_2_ NPs as a component of the sunscreen-technology revolution

During recent decades, skin cancer has become the most frequent neoplastic disease among the Caucasian population in Europe, North America and Australia, and its incidence has reached epidemic proportions.^57^ As a consequence, the trend in sun protection in daily cosmetics is towards increased use of organic and inorganic UV filters. It is estimated that worldwide use of nano-sized TiO_2_ in sunscreens is around 1000 tons per year.^51^

TiO_2_ has been used in sunscreens since 1952, however the Food and Drug Administration (FDA) approved the use of TiO_2_ in sunscreens in 1999.^58^,^59^ Currently it is not required to label sunscreens as containing nano-TiO_2._ 60 The situation could change if the European Union (EU) commission adopts a proposed new regulation within EU Cosmetic Directive, under which all cosmetics that contain more than 1 % w/w of NPs will have to declare it on the packaging. Since TiO_2_ is considered as low- irritating, it is the only inorganic UV filter allowed by European legislation in concentrations as high as 25 %.^61^,^62^ There is also some confusion regarding the classification of sunscreens. In the EU they are classified as cosmetics, while in the USA, they are classified as over the counter (OTC) drugs.^63^

The average size of the TiO_2_ particles in sun-screens ranges between 10 and 100 nm, while some products contain particles down to 5 nm or up to 500 nm.^64^ TiO_2_ particles in the size range between 200 and 500 nm are opaque and act as a true sun-block when applied to the skin.^61^,^65^,^66^ However, this opacity is lost when much finer particles are used. Such sunscreens are more transparent, less viscous, and blend into the skin more easily. Therefore, the optimum size of TiO_2_ particles was suggested to be around 50 nm, which provides good protection against UV light, while the dispersion of visible light is such that sunscreens do not appear white on the skin.^67^

**FIGURE 2:**
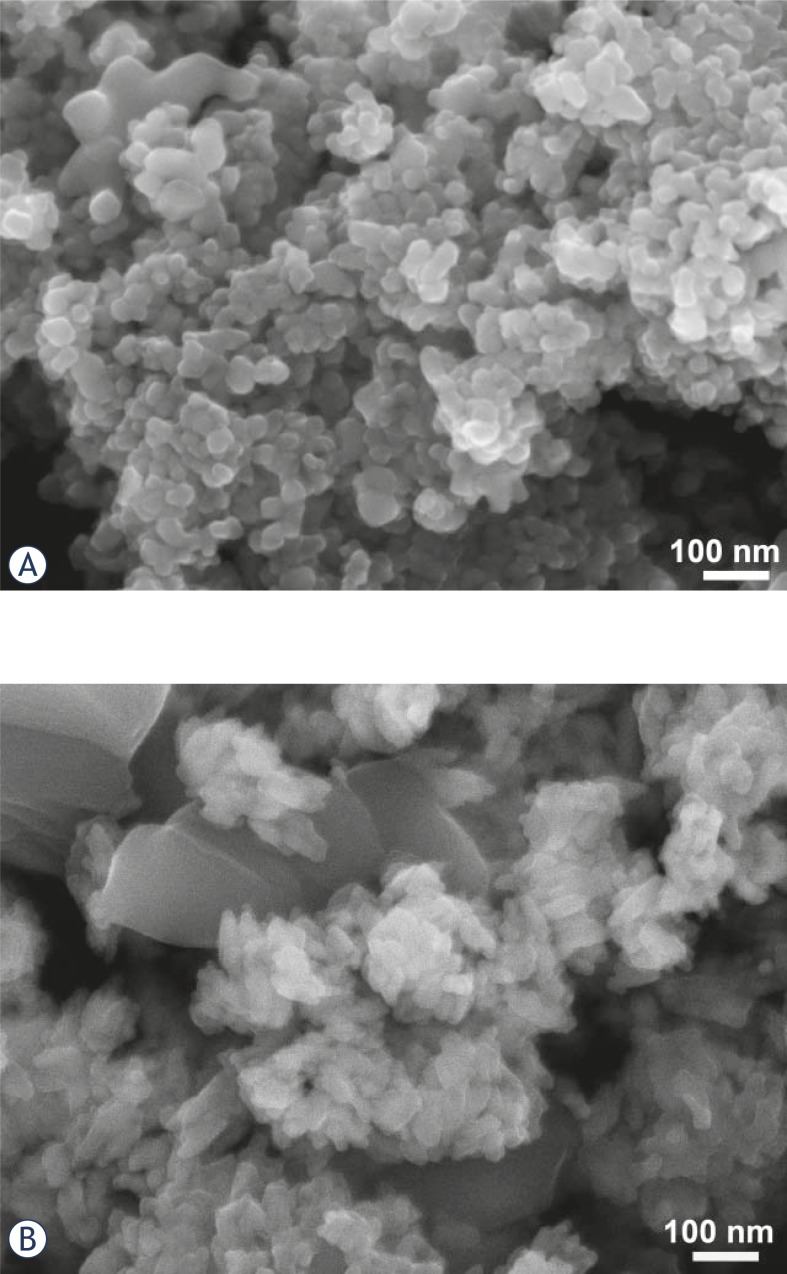

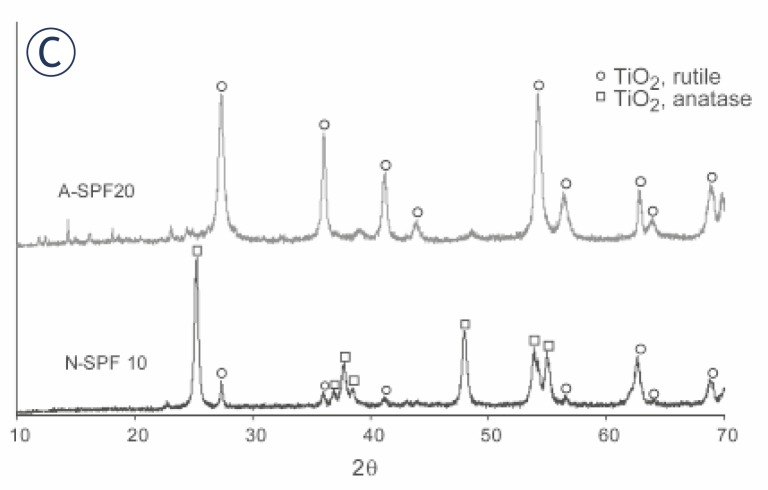
Field emission electron micrographs of the powders from two commercial sunscreens: A-SPF 20 (A) and N-SPF 10 (B), and their XRD diffraction (C).

Sunscreens typically, but not exclusively, contain rutile TiO_2_ powder, which is less photo-active than the anatase TiO_2_. Micrographs of the powders extracted from two commercial sunscreens from different producers are shown in Figures 2A and B. From the X-ray diffractograms (Figure 2C) it is evident that the TiO_2_ powder in the sunscreen “N-SPF10” is predominantly in the anatase form, with an estimated particle size of around 50 nm (Figure 2A, C), while the powder in the sunscreen “A-SPF20” contained rutile TiO_2_ with two size populations (Figure 2B, C). (The original commercial names of the products were adapted for this study.)

**FIGURE 3:**
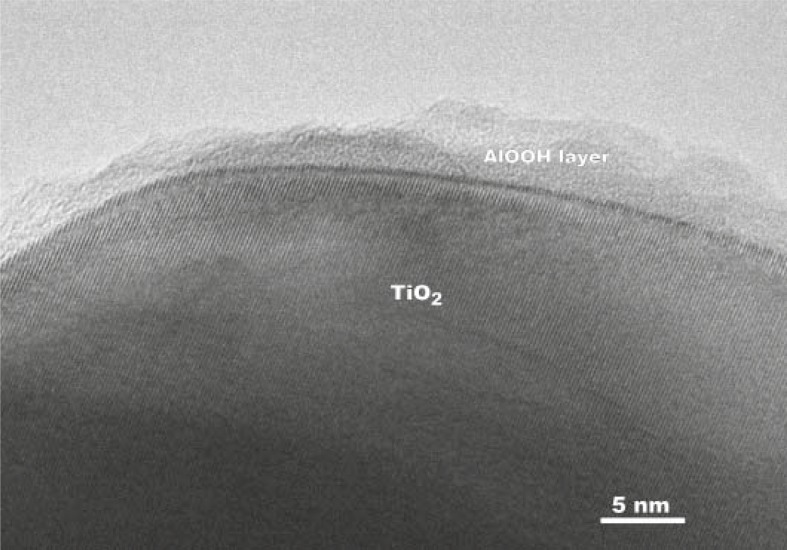
Transmission electron micrograph of an AlOOH-coated TiO_2_ NP (Courtesy of dr. G. Dražić).

To minimize the harmful effects of photo-active nano-TiO_2_, various coatings such as magnesia, silica, alumina or zirconia^68^–^71^ were introduced. However, certain coating materials may have side effects, such as aluminium-based ones (Figure 3), and it is also not clear how stable the coatings are and what is the lifetime of the “inert” particle released from sunscreens.

### Cytotoxic and genotoxic effects of TiO_2_ NPs in dermal cells and skin models

Different dermal cell types have been reported to differ in their sensitivity to nano-sized TiO_2_ . Kiss *et al*.^72^ exposed human keratinocytes (HaCaT), human dermal fibroblast cells, sebaceous gland cells (SZ95) and primary human melanocytes to 9 nm-sized TiO_2_ particles at concentrations from 0.15 to 15 μg/cm^2^ for up to 4 days. The particles were detected in the cytoplasm and perinuclear region in fibroblasts and melanocytes, but not in kerati-nocytes or sebaceous cells. The uptake was associated with an increase in the intracellular Ca^2+^ concentration. A dose- and time-dependent decrease in cell proliferation was evident in all cell types, whereas in fibroblasts an increase in cell death via apoptosis has also been observed. Anatase TiO_2_ in 20–100 nm-sized form has been shown to be cytotoxic in mouse L929 fibroblasts.^73^ The decrease in cell viability was associated with an increase in the production of ROS and the depletion of glutathione. The particles were internalized and detected within lysosomes. In human keratinocytes exposed for 24 h to non-illuminated, 7 nm-sized anatase TiO_2,_ a cluster analysis of the gene expression revealed that genes involved in the “inflammatory response” and “cell adhesion”, but not those involved in “oxidative stress” and “apoptosis”, were up-regulated.^73^ The results suggest that non-illuminated TiO_2_ particles have no significant impact on ROS-associated oxidative damage, but affect the cell-matrix adhesion in keratinocytes in extracellular matrix remodelling. In human keratinocytes, Kocbek *et al*.^19^ investigated the adverse effects of 25 nm-sized anatase TiO_2_ (5 and 10 μg/ml) after 3 months of exposure and found no changes in the cell growth and morphology, mitochondrial function and cell cycle distribution. The only change was a larger number of nanotubular intracellular connections in TiO_2_-exposed cells compared to non-exposed cells. Although the authors proposed that this change may indicate a cellular transformation, the significance of this finding is not clear. On the other hand, Dunford *et al*.^23^ studied the genotoxicity of UV-irradiated TiO_2_ extracted from sunscreen lotions, and reported severe damage to plasmid and nuclear DNA in human fibroblasts. Manitol (antioxidant) prevented DNA damage, implying that the genotoxicity was mediated by ROS.

Recently, Yanagisawa *et al.*^74^ reported that the transdermal exposure (mimicking skin-barrier dysfunction or defect) of NC/Nga mice to TiO_2_ NPs (15, 50, or 100 nm), in combination with allergen, aggravated atopic dermatitis-like lesions through a T-helper type 2 (Th2) dominant immune response. The study also indicated that TiO_2_ NPs can play a role in the initiation and/or progression of skin diseases, since histamine was released, even in the absence of allergen.

#### Skin-penetration studies

The skin of an adult person is, in most places, covered with a relatively thick (∼10 μm) barrier of keratinised dead cells. One of the main questions is still whether TiO_2_ NPs are able to penetrate into the deeper layers of the skin.^75^ The majority of studies suggest that TiO_2_ NPs, neither uncoated nor coated (SiO_2_, Al_2_O_3_ and SiO_2_/Al_2_O_3_) of different crystalline structures, penetrate normal animal or human skin.^76^,^77^–^82^ However, in most of these studies the exposures were short term (up to 48 h); only few long-term or repeated exposure studies have been published. Wu *et al.*83 have shown that dermal application of nano-TiO_2_ of different crystal structures and sizes (4–90 nm) to pig ears for 30 days did not result in penetration of NPs beyond deep epidermis. On the other hand, in the same study the authors reported dermal penetration of TiO_2_ NPs with subsequent appearance of lesions in multiple organs in hairless mice, that were dermal exposed to nano-TiO_2_ for 60 days. However, the relevance of this study for human exposure is not conclusive because hairless mice skin has abnormal hair follicles, and mice stratum corneum has higher lipid content than human stratum corneum, which may contribute to different penetration. Recently Sadrieh *et al.*^84^ performed a 4 week dermal exposure to three different TiO_2_ particles (uncoated submicron-sized, uncoated nano-sized and coated nano-sized) in 5 % sunscreen formulation with minipigs. They found elevated titanium levels in epidermis, dermis and in inguinal lymph nodes, but not in precapsular and submandibular lymph nodes and in liver. With the energy dispersive X-ray spectrometry and transmission electron microscopy (TEM) analysis the authors confirmed presence of few TiO_2_ particles in dermis and calculated that uncoated nano-sized TiO_2_ particles observed in dermis represented only 0.00008 % of the total applied amount of TiO_2_ particles. Based on the same assumptions used by the authors in their calculations it can be calculated that the total number of particles applied was 1.8 × 10^13^ /cm^2^ and of these 1.4 x10^7^/cm^2^ penetrated. The surface area of skin in humans is around 1.8 m^2^ 85 and for sun protection the cream is applied over whole body, which would mean that 4 week usage of such cream with 5 % TiO_2_ would result in penetration of totally 2.6 × 10^10^ particles. Although Sadrieh *et al*.^84^ concluded that there was no significant penetration of TiO_2_ NPs through intact normal epidermis, the results are not completely confirmative.

### TiO_2_ NPs intake by food

TiO_2_ has been well accepted in the food industry and can be found as the E171 additive in various food products, mainly for whitening and texture. It is present in some cottage and Mozzarella cheeses, horseradish cream and sauces, lemon curd, and in low-fat products such as skimmed milk and ice-cream. Even if the product is labelled as containing E171, no information is usually given about the quantity, particle size and particle structure. FDA claims that TiO_2_ may be safely used as a colour additive for colouring foods in quantities up to 1 % by weight of the food.^86^ Interestingly, TiO_2_ is frequently declared as a “natural colouring agent” and is therefore well accepted by consumers.

TiO_2_ is also used in oral pharmaceutical formulations^5^, and the Pharmaceutical Excipients handbook considers nano-sized TiO_2_ a non-irritant and non-toxic excipient. Despite the fact that TiO_2_ submicron- and nano-sized particles are widely used as food and pharmaceutical additives, information on their toxicity and distribution upon oral exposure is very limited.

#### Potential hazards of oral exposure to TiO_2_ NPs

The gastrointestinal tract is a complex barrier/exchange system, and is the most important route by which macromolecules can enter the body. The main absorption takes place through villi and microvilli of the epithelium of the small and large intestines, which have an overall surface of about 200 m^2^. Already in 1922, it was recognized by Kumagai^87^, that particles can translocate from the lumen of the intestinal tract via aggregation of intestinal lymphatic tissue (Peyer’s patch, containing M-cells (phagocytic enterocytes)). Uptake can also occur via the normal intestinal enterocytes. Solid particles, once in the sub-mucosal tissue, are able to enter both the lymphatic and blood circulation.

In an early study Jani *et al*.^88^ administred rutile TiO_2_ (500 nm) as a 0.1 ml of 2.5 % w/v suspension (12.5 mg/kg BW) to female Sprague Dawley rats, by oral gavage daily for 10 days and detected presence of particles in all the major gut associated lymphoid tissue as well as in distant organs such as the liver, spleen, lung and peritoneal tissue, but not in heart and kidney. The distribution and toxicity of nano- (25 nm, 80 nm) and submicron-sized (155 nm) TiO_2_ particles were evaluated in mice administered a large, single, oral dosing (5 g/kg BW) by gavage.^89^ In the animals that were sacrificed two weeks later, ICP-MS analysis showed that the particles were retained mainly in liver, spleen, kidney, and lung tissues, indicating that they can be transported to other tissues and organs after uptake by the gastrointestinal tract. Interestingly, although an extremely high dose was administrated, no acute toxicity was observed. In groups exposed to 80 nm and 155 nm particles, histopathological changes were observed in the liver, kidney and in the brain. The biochemical serum parameters also indicated liver, kidney and cardiovascular damage and were higher in mice treated with nano-sized (25 or 80 nm) TiO_2_ compared to submicron-sized (155 nm) TiO_2_. However, the main weaknesses of this study are the use of extremely high single dose and insufficient characterisation of the particles.

Duan *et al*.^90^ administered 125 mg/kg BW or 250 mg/kg BW of anatase TiO_2_ (5 nm) intragastrically to mice continuously for 30 days. The exposed mice lost body weight, whereas the relative liver, kidney, spleen and thymus weights increased. Particles seriously affected the haemostasis of the blood and the immune system. The decrease in the immune response could be the result of damage to the spleen, which is the largest immune organ in animals and plays an important role in the immune response. Powel *et al*.^91^ demonstrated that TiO_2_ NPs may trigger immune reactions of the intestine after oral intake. They showed that TiO_2_ NPs conjugated with bacterial lipopolysaccharide, but not TiO_2_ NPs or lipopolysaccharide alone, trigger the immune response in human peripheral blood mononuclear cells and in isolated intestinal tissue. This indicates that TiO_2_ NPs may be important mediators in overcoming normal gut-cell hyporesponsiveness to endogenous luminal molecules, which may be particularly relevant to patients with inflammatory bowel disease, which is characterized by an abnormal intestinal permeability.

The National Cancer Institute tested TiO_2_ for possible carcinogenicity by the oral route of exposure by feeding rats and mice with TiO_2_ (size not specified) at doses 25,000 or 50,000 ppm TiO_2_ for 103 weeks. They concluded that TiO_2_ was not carcinogenic.^92^ Also, the study with rats fed diets containing up to 5 % TiO_2_ coated mica for 130 weeks showed no treatment-related carcinogenicity.^93^ Since the size and other TiO_2_ properties were not specified or determined, we cannot generalize this conclusion and we have to take into account other possible outcomes of this scenario in different exposure conditions (other size/crystalline structure of TiO_2_ etc.).

It should also be considered that due to the low pH in the stomach, the increased dissolution of the TiO_2_ particles may increase its bioavailability and may facilitate the entry of titanium ions into the blood circulation.^94^ Despite the relatively large consumption of TiO_2_ as a food additive, no studies on the effect of pH on its absorption and bioavailability have been found in the literature. This can be attributed to a general belief that TiO_2_is completely insoluble. However, this is not completely true, as TiO_2_ particles show a certain degree of solubility.^33^

### Exposure to TiO_2_ NPs by inhalation

Inhalation exposure to TiO_2_ particles occurs pre-dominantly in occupational settings during production of TiO_2_ powders and manufacturing the products containing TiO_2_.^95^ The highest levels of exposure occur during packing, milling and site cleaning however, the empirical data regarding air-borne TiO_2_ particle concentrations in occupational settings is very limited. Fryzek *et al*.^96^ reported that packers, micronizers and addbackes had the highest TiO_2_ exposure levels measuring 6.2±9.4 mg/m^3^, whereas ore handlers had lower TiO_2_ exposure level of 1.1±1.1 mg/m^3^. Boffetta *et al*.^97^ reported that the yearly averaged estimated exposure to TiO_2_ dust in EU factories varied from 0.1 to 1.0 mg/m^3^, and the average levels ranged up to 5 mg/m^3^ for individual job categories. However, in these studies the particle size distribution has not been determined. Nevertheless, the data indicate that in certain jobs categories the exposure exceed the values of time-weighted average (10 h TWA) concentrations of 2.4 mg/m^3^ for submicron-sized TiO_2_ and 0.3 mg/m^3^ for nano-sized TiO_2_, which are recommended as exposure limits by National Institute for Occupational Safety and Health (NIOSH).^98^

#### Potential hazards of inhalation exposure to TiO_2_ NPs

The lung consists of about 2300 km of airways and 300 million alveoli. The epithelium of airways is protected by a viscous layer of mucus, and is a relatively robust barrier. In alveoli, the barrier between the alveolar wall and the capillaries is very thin, about 0.5 μm. Thus, the large surface area of the alveoli and the intense air-blood contact in this region makes the alveoli less protected against environmental damage than other parts of the respiratory system.^75^ The clearance of particles from the upper airways is achieved through the mucociliary escalator, while clearance from the deep lung is supposed to be achieved predominantly by macrophage phagocytosis. Deposited particles can lead to the activation of cytokine production and inflammation by macrophages and epithelial cells. It has been reported that besides the pulmonary and systemic inflammation, inhaled insoluble NPs can also accelerate atherosclerosis and alter the cardiac autonomic function.^99^–^102^

Following administration of nano-sized TiO_2_ to rats by inhalation the particles were detected in the cytoplasm of all lung-cell types in a non-membrane bound manner.^17^ Ferin *et al.*^103^ reported that 20 nm- sized TiO_2_ particles penetrate more easily into the pulmonary interstitial space of rats than 250 nm-sized TiO_2_ particles. Three-month inhalation exposure in rats demonstrated that the clearance of 20 nm TiO_2_ particles was significantly slower than that of 200 nm TiO_2_ particles, and more particles translocated to interstitial sites and regional lymph nodes.^104^ Geiser *et al.*^14^ confirmed that alveolar macrophages were not primarily responsible for the uptake and clearance of TiO_2_ NPs. These findings are in agreement with the known size limitations of uptake processes such as phagocytosis, which is thought to be restricted to particles that are 1 to 5 μm in size, while NPs might escape macrophage phagocytosis.^101^,^105^

Inhaled TiO_2_ NPs can enter the alveoli of the lung and consequently the blood circulation^106^,^107^ and can then translocate to other organs.^102^,^108^,^109^ In addition to several reports on the absence of toxicity following the inhalation of TiO_2_ NPs in rodents, the majority of lung-inhalation and instillation studies have pointed out obvious toxic effects, like inflammation and damage to pulmonary epithelium.^110^ The studies also showed that TiO_2_ NPs induced greater pulmonary inflammation and tissue damage than an equal dose of submicron-sized TiO_2_ particles. The greater toxicity of TiO_2_ NPs has been explained as being related to their larger surface area and their increased internalization.^111^ Multiple studies showed the reversibility of the inflammatory response after cessation of the exposure to TiO_2_ particles. After a single instillation exposure to different types of submicron- and nano-sized TiO_2_, acute inflammatory response returned to control levels within one week^112^ or 90 days^113^ after the instillation. In mice that were exposed to TiO_2_ NPs (2–5 nm) by whole body inhalation (0.77 or 7.22 mg/m^3^ 4 h/day 10 days) the recovery was observed during the third week after exposure.^114^

Pulmonary toxicity studies suggest that, besides the particle size and surface area, crystal structure and surface treatment are also important parameters. Warheit *et al*.^115^ demonstrated higher pulmonary toxicity of anatase than rutile TiO_2_ NPs. These observations were confirmed in a recent study by Roursgaard *et al*.^116^ who showed that the intrat-racheal instillation of submicron- and nano-sized rutile, nano-sized anatase, or amorphous TiO_2_ to mice induced a dose-dependent acute inflammation, while subchronic inflammation was apparent only in mice exposed to nano-sized rutile and amorphous TiO_2_.

Recently, toxicogenomic studies were published that may contribute to a better understanding of the mechanisms of TiO_2_-mediated pulmonary toxicity. In mice exposed to a single intratracheal dose (0.1 or 0.5 mg/kg BW) of TiO_2_ with an average particle size of 20 nm Chen *et al.*^117^ showed that changes in the morphology and histology of the lungs were associated with the differential expression of hundreds of genes, including those involved in cell cycle regulation, apoptosis, chemokines, and complement cascades. In particular, TiO_2_ NPs upregulated the expression of the placenta growth factor and other chemokines that are associated with pulmonary emphysema and alveolar epithelial cell apoptosis. Park *et al*.^118^ showed that exposure of mice to nano-sized TiO_2_ (5–50 mg /kg BW) by a single intratracheal instillation can, in addition to chronic inflammation, also trigger an autoimmune response. They found that many classes of genes related to antigen presentation and the induction of chemotaxis of immune cells were over-expressed.

The studies have shown that submicron-sized TiO_2_ 119 and nano-sized TiO_2_ 120,121 induce lung tumors in chronically exposed rats. TiO_2_ NPs induced a significantly increased number of lung tumors during inhalation exposure to 10 mg/m^3^ (18 h/day, 2 years), while submicron-sized TiO_2_ increased the number of lung tumors at exposure to 250 mg/ m^3^ (6 h/day 2 years). In contrast, no tumours were observed in similarly exposed mice and hamsters.^121^,^122^ These apparent species differences suggest that the experimentally induced lung tumours may be a rat-specific, threshold phenomenon, depending on lung overloading accompanied by chronic inflammation to exert the observed tumorigenic response. Comparative toxicological studies of the development and possible progression of the lung response in rats, mice and hamsters exposed to a range of concentrations of submicron- or nano-sized TiO_2_ over a period of 90 days showed distinct species differences in the lung responses. Rats and mice had similar lung burdens and clearance rates, while hamsters showed higher clearance rates. At high lung-particle burdens, rats showed a marked progression of the histopathological lesions during the post-exposure period, while mice and hamsters showed minimal initial lesions with apparent recovery during the post-exposure period.^123^,^124^ It has been thus argued that the dose response data from inhalation studies in rats should not be used when extrapolating the cancer risk to humans.^95^ However, clearance of insoluble particles is in humans slower than in rats.^125^ In addition, it has been shown that the lung-tumour response to exposure to non-soluble particles can be predicted by the particle surface area dose without the need to account for overloading.^98^ Therefore, for workers with a high dust exposure the doses that cause overloading in rats may be relevant for estimating the health risk for humans.

Animal studies showed also other adverse effects after inhalation exposure to TiO_2_ particles. Nurkiewicz *et al*.^109^ showed that exposure to TiO_2_ particles may cause cardiovascular effects at concentrations below those causing adverse pulmonary effects. In rats exposed to submicron-sized TiO_2_ m) or nano-sized TiO_2_ (<1 μ (21 nm) at airborne exposures aimed at achieving similar particle mass deposition in the lungs (nano-sized: 1.5–12 mg/ m^3^, 240–720 min; submicron-sized: 3–15 mg/m^3^, 240–480 min) they observed systemic microvessel dysfunction in the absence of pulmonary inflammation or lung damage. The effect was related to the adherence of polymorphonuclear leukocytes to the microvessel walls and the production of ROS in the microvessels. As already described previously, inhalation exposure to TiO_2_ NPs may cause immune responses and neurotoxic effects that may lead to respiratory allergy and higher risk of developing asthma, respectively.

It has been reported that TiO_2_ NPs can translocate to the central nervous system following nasal instillation, potentially via the olfactory bulb, and accumulate mainly within the cerebral cortex, thalamus and hippocampus.^22^,^29^,^126^ The absorption appears to occur via neuronal transport, bypassing the blood-brain barrier.^29^,^126^ The main target is the hippocampus, where TiO_2_ NPs caused morphological alteration and the loss of neurones. In addition, TiO_2_ induced oxidative stress and an inflammatory response within the whole brain, with anatase nano-TiO_2_ inducing a stronger inflammatory response than rutile. However, from these studies it is not clear to what extent large local doses during nasal instillation reflect inhalation exposure.

#### Human epidemiological studies

Several case reports described adverse health effects in workers with potential TiO_2_ exposure that later lead to epidemiological studies of a relationship between occupational exposure and observed cases.^98^ The lung particle analyses indicated that workers exposed to respirable TiO_2_ had particle retention in their lungs that included TiO_2_, silica, and other minerals, sometimes years after cessation of exposure. In most cases of tissue-deposited TiO_2_ was associated with a local macrophage response and fibrosis that was generally mild. In one case papillary adenocarcinoma and TiO_2_ associated pneumoconiosis was reported in the lung of a 53-year-old male who had been engaged in packing TiO_2_ for about 13 years and had 40-year smoking history.^127^ The cohort epidemiological studies undertaken in the USA 96,128 did not report excess risks of lung cancer; nor did a Canadian population-based case-control study.^129^ The retrospective cohort lung cancer mortality study^130^, which included workers in the TiO_2_ production industry in six European countries, showed a small but significant elevation in lung cancer mortality among male TiO_2_ workers when compared to the general population. However, the data did not suggest an exposure-response relation.

TiO_2_ has been classified by the International Agency for Research on Cancer (IARC) as an IARC Group 2B carcinogen, “possibly carcinogenic to humans” by inhalation.^131^ Although the IARC working group concluded that the epidemiological studies on TiO_2_ provide inadequate evidence of carcinogenicity, they considered that the results from animal studies of inhalation and intratracheal instillation provide sufficient evidence to classify TiO_2_ in Group 2B.^132^ Also NIOSH^98^ has recently classified TiO_2_ NPs as a potential occupational carcinogen but considered that there is insufficient evidence at this time to classify also submicron-sized TiO_2_ as a potential occupational carcinogen. NIOSH also recommended new exposure limits at 2.4 mg/m^3^ for submicron-sized TiO_2_ and 0.3 mg/m^3^ for nano-sized TiO_2_, as time-weighted average concentrations for up to 10 hours per day during a 40-hour work week.

### Exposure to TiO_2_ NPs through body implants

A few-nanometres-thick layer of amorphous TiO_2_ is commonly formed on the surface of orthopaedic and dental implants made of titanium metal or its alloys. In non-moving implants (hip stems, plates, screws, etc.) this does not appear to represent the same kind of risk for the body as free TiO_2_ NPs discussed in previous sections. However, this is not the case for wear-exposed implants, such as hip and knee joints. There are many reports proving that under mechanical stress or altered physiological conditions, Ti-based implants can release biologically relevant amounts of debris, in both the micrometre and nanometre ranges, that can migrate to the surrounding tissues. During the wear process, a thin amorphous oxide layer is continuously being created and removed, resulting in large numbers of titanium particles. It is increasingly being suggested that they are associated with major inflammation and systemic diseases.^133^ Furthermore, increasing numbers of reports indicate that the delayed hypersensitivity to titanium and its oxides may constitute a health risk for individuals with higher susceptibility.^134^–^136^

The effects of the TiO_2_ particles released from implants were investigated by Wang *et al.*^137^ in rats by intra-articular injection of 0.2 to 20 mg of anatase nano-TiO_2_ per kg BW. Their results demonstrate that particles can potentially affect major organs like the heart, lung and liver. Generally, the maximum diameter of particles that move across the synovial capillary wall was suggested to be 50 nm. The released TiO_2_ NPs resulted in synovial hypotrophy, lymphocyte and plasma infiltration, and fibroblast proliferation in the knee joint. Oxidative stress and lipid peroxidation was detected in exposed synovial fluid. Seven days after the initial exposure a brown particulate deposit was observed in vascular endothelial cells and in alveolar macrophages. Similar results have been reported by Urban *et al*.^138^, who found TiO_2_ particles in the liver and in the spleen of the patients with implants. TiO_2_ NPs were observed in joint simulators and in joint periprosthetic tissues. Margevicius *et al*.^139^ characterized the debris around the total hip joint prosthesis and found up to 140.10^9^ particles/g dry weight, in diameters ranging from 0.58 to 100 μm. Agins *et al*.^140^ found concentration of wear particles in the tissue adjacent to a prosthesis in the range between 56 μg/g and 3.7 mg/g dry weight. Thus, due to the natural tendency of titanium to oxidise, Ti-based implants should not be neglected as a possible source of TiO_2_ exposure.

On the other hand, the man-made (crystalline) TiO_2_ coatings on the surfaces of pure Ti or Ti alloys are reported to be able to modulate protein absorption, cell adhesion, osseointegration and bone mineralization at the bone-biomaterial interface, both *in vivo* and *in vitro.*^141^,^142^ For this reason, the development of a more stable crystalline titania coating on Ti-based implants is in progress.^143^

## Environmental pollution by TiO_2_ NPs

### Toxic effects of TiO_2_ NPs on aquatic organisms

The trend in the production of NPs is likely to lead to increasing amounts of nano-powders in the air, water and soil, which will consequently affect living organisms. Labielle *et al.*^68^ demonstrated that 25 % of Al(OH)_3_-coated TiO_2_ particles from sunscreens are dispersed as a stable colloid and become available to microorganisms and filter-feeders, while the remaining 75 % are probably incorporated into geogenic sediments, where they could become available to benthic fauna. Solar UV iradiation may penetrate as far as 20 m in the water column 144 and therefore photo-activate the dispersed particles, which may have an adverse effect on various aquatic organisms.

Freshwater algae show low-to-moderate susceptibility to TiO_2_ exposure, with more pronounced toxic effects in the presence of UV irradiation. It has also been shown that nano-sized TiO_2_ is significantly more toxic to algae *Pseudokirchneriella sub-capitata* than submicron-sized TiO_2_.^145^ Hund-Rinke and Simon 146 reported that UV irradiated 25 nm TiO_2_ NPs are more toxic to green freshwater algae *Desmodesmus subspicatus* than UV irradiated 50 nm particles, which is in agreement with Hartmann *et al*.^147^ UV irradiated TiO_2_ NPs also inactivated other algae species such as *Anabaena*, *Microcystis*, *Melsoira*^148^ and *Chroococcus.*^149^ It was demonstrated that smaller particles have a greater potential to penetrate the cell interior than submicron-sized particles and larger aggregates. Studies have shown that the amount of TiO_2_ adsorbed on algal cells can be up to 2.3 times their own weight.^142^

Nano-sized TiO_2_ generally shows low or no acute toxicity in both invertebrates^146^ and vertebrates.^150^ However, exposure of *Daphnia magna* to 20 ppm TiO_2_ for 8 consecutive days was found to cause 40 % mortality.^151^ Zhu *et al.*^152^ showed minimal toxicity to *D. magna* after 48 h exposure, while upon chronic exposure for 21 days, *D. magna* suffered severe growth retardation and mortality. A significant amount of nano-sized TiO_2_ was found also accumulated in the body of the animals. Similar findings with coated nano-sized TiO_2_ (T-Lite™ SF, T-Lite™ SF-S and T-Lite™ MAX; BASF SE) were reported by Wiench *et al*.^153^ Biochemical measurements showed that exposure to TiO_2_ NPs induces significant concentration-dependent antioxidant enzyme activities in *D. magna*^154^. Lee *et al*.^155^ showed that 7 and 20 nm-sized TiO_2_ induced no genotoxic effect in *D. magna* and in the larva of the aquatic midge *Chironomus riparius.*

No acute effects of nano-sized TiO_2_ were observed in *Danio rerio* (zebrafish) embryos.^156^ Exposure of rainbow trout to TiO_2_ NPs triggered lipid peroxidation, influence on the respiratory tract, disturbance in the metabolism of Cu and Zn, induction of intestinal erosion 157 and accumulation in kidney tissue.^158^ Linhua *et al.*^159^ exposed juvenile carp to 100 and 200 mg/ml of particles and TiO_2_ observed no mortality. However, the fish suffered from oxidative stress and pathological changes in gill and liver. In the infaunal species *Arenicola marina*, exposure to TiO_2_ NPs in sediment caused sub-lethal effects including decrease in casting rate and increase in cellular and DNA damage.^160^ Aggregated particles were visible in the lumen of the gut, but no uptake through the gut or the skin was observed.

Zhu *et al*.^161^ were the first to provide evidence that TiO_2_ NPs (21 nm) can transfer from daphnia to zebrafish by dietary exposure. Hence, dietary intake could be a major route of exposure to NPs for high trophic level aquatic organisms. Ecological research should therefore focus, not only on the concentration of NPs in the environment, but also on its bioconcentration, bioaccumulation and biomagnification. In addition it has been shown that TiO_2_ NPs can increase accumulation of other environmental toxicants: enhanced accumulation of cadmium (Cd) and arsenic (As) was found in carp in the presence of TiO_2_ NPs.^162^,^163^ The strong adsorption capacity for Cd and As was explained by the large specific surface area and strong electrostatic attraction of TiO_2_ NPs that contribute to facilitated transport into different organs.

*In vitro*, in the hemocytes of the marine mussel *Mytilus hemocytes*, suspension of TiO_2_ NPs (Degussa P25, 10 μg/ml) stimulated immune and inflammatory responses, such as lysozyme release, oxidative burst and nitric oxide production.^164^ Vevers and Jha^165^ demonstrated the intrinsic genotoxic and cytotoxic potential of TiO_2_ NPs on a fish-cell line derived from rainbow-trout gonadal tissue (RTG-2 cells) after 24 h of exposure to 50 μg/ml. Reeves *et al.*^166^ demonstrated a significant increase in the level of oxidative DNA damage in goldfish cells, and suggested that damage could not repaired by DNA repair mechanisms. Another suggestion from the mentioned study was that hydroxyl radicals are generated also in the absence of UV light. It has been shown that fish cells are generally more susceptible to toxic/oxidative injury than mammalian cells.

### Toxic effects of TiO_2_ NPs on soil organisms

Drobne *et al*.^167^ used the terrestrial arthropod *Porcellio scaber* as a test organism for determining the cytotoxic effect of TiO_2_ NPs (anatase). The animals were exposed to TiO_2_ NPs of two different sizes (25 nm and 75 nm) in the concentration range 10–1000 μg TiO_2_/g dry food for 3 to 14 days. No adverse effects, such as mortality, body weight changes or reduced feeding, were observed. In fact, quite the opposite, an enhanced feeding rate, food absorption efficiency and increase in catalase activity were observed. The intensity of these responses appeared to be time- but not dose-dependent. It should also be noted that the concentrations tested in this study were much higher than the predicted concentration (4.8 μg/g soil) at high emission scenario of nano-sized TiO_2_.^168^ Using the same test organism another group^169^ showed that exposure to TiO_2_ NPs induced destabilization of cell membrane in the epithelium of digestive glands isolated from exposed animals. They also showed that this effect can be observed after just 30 minutes of exposure.

TiO_2_ NPs appeared to be more toxic to nematode *Caenorhabditis elegans* than submicron-sized TiO_2._ 170 At a concentration of 1 mg/l, 7 nm particles affected its fertility and survival rate and were more toxic than 20 nm anatase particles.^171^ Similarly, Hu *et al.*^172^ showed that rutile particles (10–20 nm), at concentrations above 1 g/kg soil, can be bio-accumulated in earthworms, where they induce oxidative stress, inhibit the activity of cellulase and induce DNA and mitochondrial damage.

### The effects of TiO_2_ NPs in plants

In addition to the toxic effects of TiO_2_ NPs, discussed in previous chapters, these NPs have been also shown to promote photosynthesis and nitrogen metabolism, resulting in the enhanced growth of spinach.^173^–^175^ It increases the absorption of light and accelerates the transfer and transformation of the light energy.^176^ It was also found that treatment with nano-sized TiO_2_ significantly increased the level of antioxidant enzymes, and decreased the ROS accumulation and malonyldialdehyde content in spinach chloroplasts under visible and UV irradiation.^177^ TiO_2_ NPs also increased the superoxide dismutase activity of germinating soybean, enhanced its antioxidant ability, and promoted seed germination and seedling growth.^178^

## Potential desirable effects of TiO_2_ NPs

The same properties of nano-sized TiO_2_ that are associated with undesirable, harmful effects can be exploited for certain useful applications. The antimicrobial effect of photo-activated TiO_2_ NPs has been known since 1985^179^ and since then numerous reports have described its potential antimicrobial activity against numerous microorganisms.^180^ As expected, the antimicrobial effect increases with smaller particle sizes^181^; however, powder agglomeration may obscure this effect.^151^ When submitted to UV-C irradiation, TiO_2_ depresses the photo-activation and dark repair of DNA in bacteria, which increases the bactericidal efficiency of UV-C irradiation.^182^

TiO_2_ NPs have potential application in removing or minimizing the effect of the red tides^183^ that are associated with the harmful algae *K.brevis* that produces neurotoxic brevetoxin (PbTxs). Further, it can be used for disinfecting water, air and surfaces, with possible applications of TiO_2_ in form of solid films or free particles. Given its use for eradicating toxins, pollutants and spores from water and air, it can be classified as a broad-spectrum oxidizing/cleaning substance. However, an informed balance between the benefits of such a cleaning system and its potential adverse effects needs to be maintained.

NPs are offering new possibilities for in medicine either for diagnostic or therapeutic purposes. For instance recent studies indicate that magnetic NPs may be used in cancer treatment for targeted drug delivery.^184^ Several recent studies indicated that also cultured cancer cells are more sensitive to TiO_2_ NPs than normal cells.. Photo-activated TiO_2_ exhibited selective cytotoxicity against highly malignant breast-cancer cells MDA-MB-468, in comparison with non-malignant MCF-7 cells.^185^ Similarly, UV-irradiated Degussa P25 TiO_2_ NPs reduced viability of sarcoma cells but were not toxic to cultured fibroblasts MCR-5.^186^ In addition, UV-C photo-activated TiO_2_ particles inhibited aggregation of sarcoma cells with human platelets, thus preventing the formation of metastases. Cai *et al*.^187^ found that photo-activated (50 μg/ml), but not non-irradiated nano-sized TiO_2_, was lethal for HeLa cells *in vitro* and suppressed the growth of HeLa tumours in nude mice. Photo-activated TiO_2_ also showed antitumour activity *in vivo* against murine skin tumours.^188^ The potential usefulness of nano-sized TiO_2_ in cancer cell therapy has also been reported by other research groups.^189^–^192^ Cytotoxicity against different cancer cell lines appears to depend on the cell type, the particle concentration and the surface chemistry.

The appearance of multidrug-resistant tumour cells is a major obstacle to the success of chemotherapy. Song *et al*.^193^ reported an enhanced effect of nano-sized TiO_2_ on drug uptake by drug-resistant leukaemia cells under UV irradiation. Very promising is also the finding that cancer cells can be effectively destroyed by the use of X-ray irradiated nano-sized TiO_2_.^194^ A combination of monoclonal antibody conjugated nano-sized TiO_2_ with photoinduction^195^ and electroporation^196^ have also been proposed for selective cancer treatment. The monoclonal antibodies would enable selective targeting of cancer cells, photoinduction would trigger local generation of radicals and electroporation would accelerate the delivery of nano-sized TiO_2_ into the cancer cells. A novel possibility of cancer treatment was recently suggested^197^, in which TiO_2_ NPs and folic acid were coupled and shown to be internalized by HeLa cells via the folate receptor.

## Where we are and where to go

The mechanistic toxicological studies showed that TiO_2_ NPs induced adverse effects are predominantly mediated by oxidative stress, which may lead to cell damage, genotoxic effects, inflammatory responses and changes in cell signalling. The studies also showed that these effects strongly depend on numerous chemical and physical characteristics of the TiO_2_ particles: size, crystal structure, specific surface area, particle shape, purity, surface charge, solubility, agglomeration rate, photo-activation, etc. TiO_2_ particles are without doubt associated with the hazardous properties, and the risk for human health and environment depends on the route and extent of exposure.

Based on the widespread use of creams with SPF based on nano-sized TiO_2_, human exposure to TiO_2_ NPs by dermal applications is apparently enormous. *In vitro* studies with skin models showed that TiO_2_ NPs are taken up by keratinocytes, fibroblasts, and melanocytes, in which they cause toxic effects that are not different from the effects observed in other cell types. Current experimental evidence indicates that TiO_2_ NPs do not penetrate through healthy skin and thus do not reach viable skin cells and distribute to other organs and tissues. However, the data on TiO_2_ NPs skin penetration during long-term or repeated exposure and in the presence of UV, which is actually characteristic for real life exposure, are insufficient. Therefore, there is no simple answer to the question regarding safety of the use of TiO_2_ NPs in sunscreens. The safety of the use of TiO_2_ in cosmetics is often argumented by the claim, that it has been used for decades without observing any adverse effects on human health. This, however, is not completely true, as no monitoring and post market health surveillance has been conducted, neither for submicron-sized nor for nano-sized TiO_2_ in sunscreens. Such surveillance is currently impossible, since current legislation does not require labelling whether the products contain nano-sized TiO_2_, which is also incorrect to customers who have no possibility to make a choice whether to use or not the sunscreen containing nano-sized TiO_2_. In our opinion dermal applications of TiO_2_ NPs as sunscreen should be limited until appropriate long-term experimental studies confirm their harmlessness. It is undeniable that long-term sun exposure can induce skin cancer. It is questionable, however, whether people are, by using sunscreens, actually encouraged to expose themselves to the sun instead of avoiding it, and if the benefit provided by TiO_2_ as a protection from UV compensates for the potential harm.

The available data on absorption, distribution, elimination or any consequent adverse effects after oral exposure to specific TiO_2_ NPs are extremely limited. TiO_2_ NPs have been shown to be absorbed from gastrointestinal tract and distributed to other organs, however this was observed at extremely high, for human exposure, irrelevant doses. On the other hand, it has been shown that at lower concentrations TiO_2_ may induce different adverse effects. TiO_2_ is an approved food additive with the limit set at 1 % by weight of the food; however, neither the size nor the structure is defined. It has been estimated that the average daily exposure to TiO_2_ from food, medicines and toothpaste is around 5 mg/individual (*i.e.,* about 0.07 mg/kg BW)^198^, which is a much lower dose than those that showed adverse effects in experimental animals. Currently there is no data if, and what proportion of TiO_2_ NPs is absorbed at doses relevant for human exposure, and how different food matrices affect behaviour and absorption of TiO_2_ NPs. However, even if very small portion of consumed nano-sized TiO_2_ is absorbed from gastrointestinal tract and distributed to distant organs, this brings into question accumulation of TiO_2_ NPs that may, through a constant lifetime oral exposure, reach concentrations that would trigger adverse effects. Another important question, which should not be neglected is, whether low exposure may trigger symptoms in subjects with an underlying susceptibility. Before *in vivo* toxicokinetic data for nano-sized TiO_2_ are available, no conclusion about the risk of nano-sized TiO_2_ by oral exposure is possible. Therefore, it should be seriously reconsidered if the use of TiO_2_ NPs in nutrition and pharmacy just to shade or stabilise the products is justified at all.

Inhalation seems to be the most vulnerable entrance point of the TiO_2_ NPs and the toxic effects of inhalation exposure are therefore by far the most studied. Animal studies showed that on inhalation exposure the particles deposit in the lung, where they may cause chronic inflammation and lung-tissue damage, which can lead to lung-tumour development. The important finding is that inhalation exposure to nano-sized TiO_2_ represents a higher health risk than exposure to submicron-sized TiO_2_ particles. Experimental data indicate that on inhalation exposure nano-sized TiO_2_ may translocate to distant organs and tissues, which may be associated with systemic effects, such as allergy, asthma and cardiovascular effects, however further studies are needed to confirm these observations and to clarify if they are associated with increased risk for humans. In the scientific community there is still a debate whether the data from *in vivo* rodent toxicity studies are reliable enough to predict the effects in humans in particular regarding mode of exposure (instillation vs. inhalation exposure) and the differences in susceptibility between different experimental species. Nevertheless, the experimental evidence, although not clearly supported by human epidemiological data, was considered to be sufficient to classify TiO_2_ (unrespectable to particle size and form) as “possible human carcinogen” upon inhalation exposure by IARC. Recently also NIOSH classified nano-sized, but not submicron-sized TiO_2_ as occupational carcinogen, and accordingly established different limit values for occupational inhalation exposure for nano-sized (0.3 mg/m^3^) and submicron-sized (2.4 mg/m^3^) TiO_2_. At present, through environmental air pollution general population is probably not at risk. However, occupational exposure should be controlled and protective measures applied, not only in TiO_2_ production industries, but also in certain areas of TiO_2_ applications; for instance when removing paints or destroying TiO_2_ containing materials the workers may be exposed to high concentrations of TiO_2_. Thus, accurate, portable, and cost effective measurement techniques should be developed and applied for effective exposure control and protection.

TiO_2_ can also be released within the human body as a result of the wear of Ti-based implants. The released particles cause local inflammation, but even more importantly they distribute over the body and can potentially cause systemic effects. Generally the benefit provided by the implant compensates for the potential harm, in particular in the cases where there is no better alternative to the Ti-based implants available. However, although there is no direct experimental evidence that released TiO_2_ can be deposited in the body or can cause systemic effects, it can be postulated from other exposure studies and mechanistic data that at least for individuals with hypersensitivity to titanium such exposure may represent a permanent health threat. Thus, it should be obligatory to test the patients for titanium hypersensitivity prior to implantation of titanium based implants.

Due to the widespread use TiO_2_ can enter aquatic and terrestrial environment and potentially affect the indigenous organisms. Although data from acute ecotoxicity tests in crustaceans, fish and algae indicate a low toxic potential of TiO_2_ NPs for aquatic species, when chronic exposure was applied TiO_2_ NPs induced a range of sub-lethal adverse effects. In addition it has been shown that nano-sized TiO_2_ can enter the freshwater food chain, which means that it can be transferred from lower to higher trophic organisms, including humans.

Taken together, the overall exposure of an average individual TiO_2_ NPs is not known; there are still opened questions regarding toxicokinetics and specific organ toxicity of TiO_2_ NPs, in particular at oral and dermal exposure, and thus it is impossible to make a reliable quantitative risk assessment. One of the main observations of this review is that, due to the versatility of the TiO_2_ NPs in terms of particle size, shape, crystal structure, dispersion in biological surroundings (bioavailability) and UV-induced photocatalytic activity, no single conclusion can be drawn, since different forms of TiO_2_ may act very differently. Until we know more, in our opinion TiO_2_ NPs should be used with great care, in particular in food and cosmetics. The least that should be done for the consumer is that a declaration of nano-sized TiO_2_ in these products is obligatory, so that we will have the choice whether to use it or not.
